# Raccoon eyes as a diagnostic key for suspected amyloidosis

**DOI:** 10.1093/rap/rkad105

**Published:** 2023-11-28

**Authors:** Miguel Mansilla-Polo, Rafael Botella-Estrada

**Affiliations:** Department of Dermatology, Hospital Universitario y Politécnico La Fe, Valencia, Spain; Instituto de Investigación Sanitaria (IIS), La Fe, Valencia, Spain; Department of Dermatology, Hospital Universitario y Politécnico La Fe, Valencia, Spain; Instituto de Investigación Sanitaria (IIS), La Fe, Valencia, Spain; Universidad de Valencia, Valencia, Spain

A 74-year-old male was admitted to the emergency department with cutaneous lesions on his face resembling raccoon eyes and lesions on his neck and shoulders (Fig. 1). He had also been experiencing asthenia, anorexia, weight loss and symmetric polyarthritis for several months. Laboratory tests showed elevated acute phase reactants. Initial suspicion of amyloidosis led to further investigations, including serum and urine protein electrophoresis and immunofixation, resulting in a diagnosis of primary amyloidosis associated with IgG multiple myeloma. The patient began conventional treatment for multiple myeloma. However, he died from another condition (bacterial pneumonia) 2 months later.

**Figure 1.**
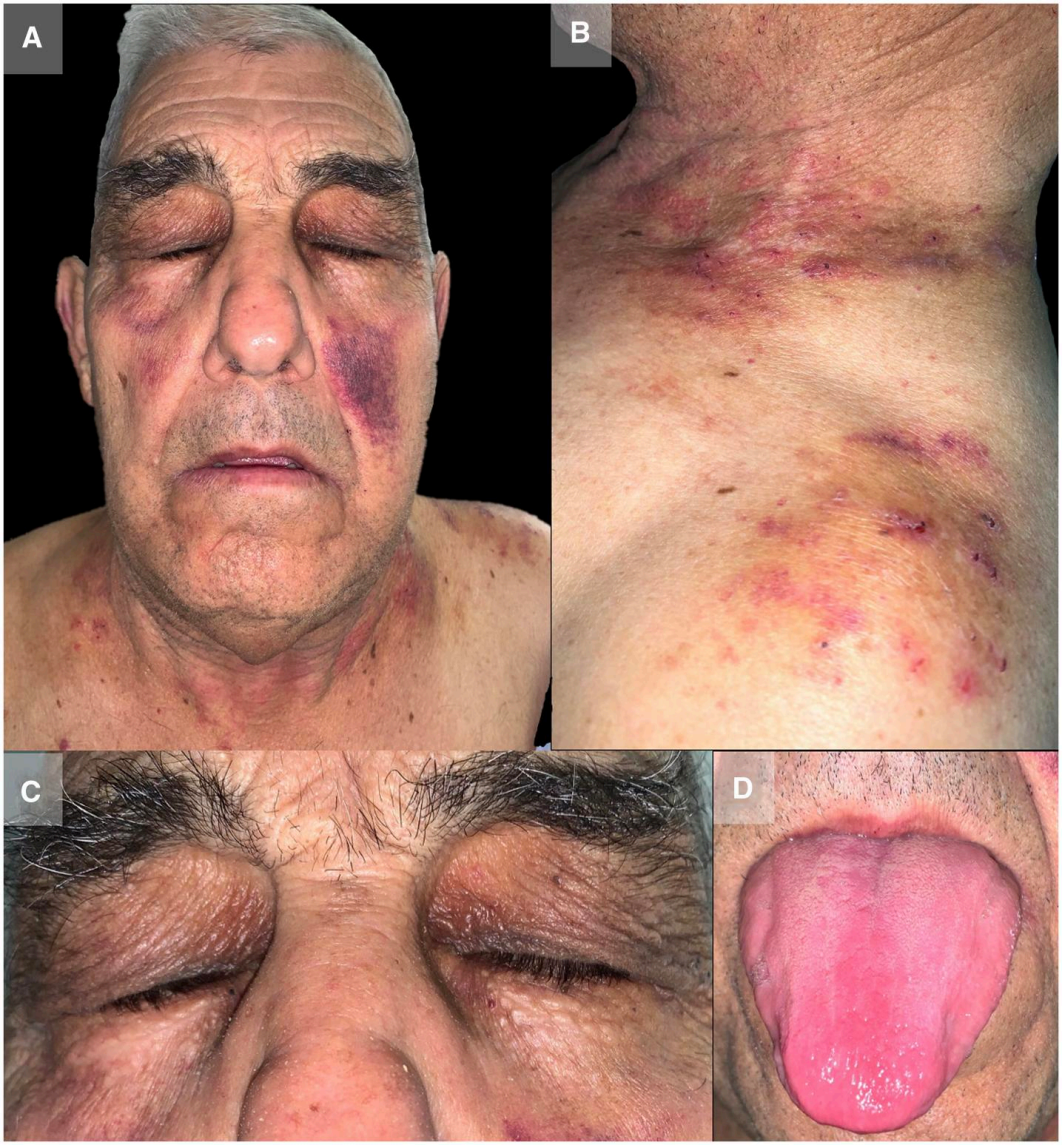
Lesions at the time of consultation. (**A–C**) Macules and ecchymotic-appearing spots on the face, neck and shoulders. (**C**) Lesions are particularly striking around the eyes, resembling raccoon eyes. (**D**) Macroglossia was observed

Primary amyloidosis involves the extracellular deposition of amyloid protein in various tissues, causing multiorgan damage [1]. Raccoon eyes, characterized by ecchymotic lesions around the eyes, are a typical cutaneous manifestation. It is essential to differentiate this from other eyelid conditions, such as atopic eczema, ocular rosacea, contact dermatitis or DM [2]. Raccoon eyes in amyloidosis result from amyloid accumulation in periorbital blood vessels and dermal laxity, leading to vascular rupture, haemorrhage and amyloid deposits [1, 2]. Recognizing raccoon eyes early is crucial for a timely diagnosis and better patient prognosis.

## Data Availability

This work has not been published or presented elsewhere in part or in entirety and a copyright transfer. All data involved during this study are included in this published article.
